# Beyond the pandemic: the relationship between macroeconomic conditions and healthcare worker shortages in the United States

**DOI:** 10.1186/s12913-025-12780-z

**Published:** 2025-05-02

**Authors:** Kyla F. Woodward, Janette Dill, LaTonya Trotter, Bianca K. Frogner

**Affiliations:** 1 Center for Health Workforce Studies, Department of Family Medicine, University of Washington, Box 354982, Seattle, WA 98195 USA; 2 Division of Health Policy and Management, University of Minnesota School of Public Health, Mayo Mail Code 197, Minneapolis, MN 55455 USA; 3 Department of Bioethics and Humanities, University of Washington, Box 357120, Seattle, WA 98195 USA

**Keywords:** Health workforce, Workforce shortages, Macroeconomic factors, Healthcare employment, Recruitment and retention

## Abstract

**Background:**

While the COVID-19 pandemic undoubtedly catalyzed healthcare workforce challenges, alone it does not explain ongoing workforce shortages. This study examines how broader economic trends and industry competition may influence worker movement in and out of healthcare. This analysis aims to (1) examine healthcare worker leaver rates over time, (2) quantify the strength of the relationship between national unemployment and healthcare worker leaver rates, and (3) describe variation in this relationship by worker characteristics.

**Methods:**

This observational, repeated cross-sectional study used nationally representative data from the 2003–2022 Annual Social and Economic Supplement of the Current Population Survey. Respondents are adult US civilian individuals employed in the healthcare industry. For each year, we defined healthcare leavers as individuals in healthcare-specific occupations who reported exiting the healthcare industry between the prior and current year, including workers who (1) changed industry, termed “industry leavers”, (2) left the labor market, or (3) became unemployed. We compared healthcare leaver rates among healthcare workers to unemployment rates for all US workers with particular focus on industry leavers. Leaver rates were analyzed by worker sociodemographic and occupational characteristics.

**Results:**

Healthcare industry leaver rates had a moderate negative correlation with the US unemployment rate (*r*=-0.58, *p =* 0.007) and unemployment rates in several competing industries of professional services (*r*=-0.61, *p* = 0.005) and retail (*r*=-0.58, *p* = 0.008), but weaker insignificant negative correlations with hospitality (*r*=-0.29, *p* = 0.215) and educational services unemployment (*r*=-0.21, *p* = 0.375). Weak to moderate negative correlations were noted between the US unemployment rate and industry leaver rates of workers in high (*r*=-0.32, *p* = 0.169) and medium (*r*=-0.52, *p* = 0.020) education groups, while industry leaver rates in low education groups had a stronger negative correlation with the unemployment rate (*r*=-0.60, *p* = 0.005).

**Conclusions:**

The easing of pandemic-era stresses on healthcare has not resolved healthcare workforce shortages. The findings of this study on historical employment trends suggest that when overall unemployment remains low, healthcare employers may struggle to recruit and retain workers due to competition from other sectors. Findings suggest that healthcare employers should take a long-range, cyclical view of worker shortages by tailoring their workforce strategies to current economic conditions while planning for the next cycle.

**Supplementary Information:**

The online version contains supplementary material available at 10.1186/s12913-025-12780-z.

## Introduction

The COVID-19 pandemic sparked national alarm about the supply of healthcare workers. While workforce researchers have projected shortages in specific occupations for the past decade [1, 2], the circumstances of the pandemic both highlighted and exacerbated significant challenges in retaining workers and filling vacancies throughout the industry. For example, skilled nursing facilities have had continual declines in employment levels since the start of the pandemic [3], and a rising share of facilities reported shortages of direct care workers (above 30%) through late 2021 [4]. Rural healthcare organizations have also faced unprecedented difficulty during the pandemic in hiring and retaining physicians and registered nurses (RNs), as well as critical support staff such as coders, schedulers, and nursing assistants [5, 6]. In early 2022, the American Hospital Association wrote to the House Energy and Commerce Committee, identifying the overall shortage of RNs and other healthcare workers as a national emergency [7].

While the pandemic was undoubtedly a catalyst for health worker scarcity, it does not explain ongoing healthcare worker shortages. Months after COVID-19 hospitalizations had decreased to non-disruptive levels, hospitals continued to report widespread problems recruiting and retaining workers [8]. In order to understand why pandemic-induced shortages seemed to have persisted after the precipitating event, we argue that there is utility in looking at more longstanding trends in healthcare workforce dynamics and their relationship to macroeconomic conditions. We know, for example, that when unemployment is low, workers may leave healthcare for other industries offering higher wages and better working conditions [9, 10]. Healthcare workers often move between jobs in healthcare, retail, educational services (e.g., academic institutions), and hospitality, with greater movement among workers in jobs such as care aides or assistants that require less education but make up nearly 40% of the health workforce [2]. In changing the incentive structure around healthcare employment, the pandemic undoubtedly shaped worker decisions, but it is unclear how much of the current problem of worker retention is due to industry-specific conditions, and how much might be explained by larger, macroeconomic conditions.

When we look at trends prior to the pandemic, we see a specific relationship between worker supply and national unemployment rates. Healthcare is one of the few industries that has shown a countercyclical relationship between industry-specific and national unemployment rates, such that healthcare employment increases when jobs are lost in the broader economy and decreases when jobs are gained. This countercyclical relationship was most pronounced during the Great Recession (2007–2009). During that time, the national employment decreased by 6.9% (7.8 million jobs) while healthcare employment increased by 6.6% (850,000 jobs) [11]. In other words, the Great Recession contributed to an increased supply of workers employed in the healthcare industry. This countercyclical trend was also evident at the local level, where healthcare employment increased more in areas experiencing severe local economic downturns compared to areas with less severe downturns [12]. Another signal of this countercyclical relationship was the disproportionate wage increase for healthcare workers (7.8%) compared to the national average wage increase (4.7%) during the Great Recession [11].

The countercyclical relationship between macroeconomic and healthcare trends suggests that the healthcare sector serves as an important stabilizing force to the economy [13, 14]. Although the mechanisms of this stabilization are not entirely known, contributing factors may include workers returning to the workforce after extended absences, increasing their hours or delaying retirement to meet the needs of their families, or lacking opportunity to work outside of the healthcare sector as the economy contracted [13, 15, 16]. These opportunities and decisions may also be impacted by individual gender and race. Previous research provides robust evidence that women disproportionally bear the burden of childcare and eldercare, which put them at greater risk of leaving the labor market throughout their working lives [17], and the pandemic only served to widen these gender disparities [18, 19]. Research on employment among underrepresented racial or ethnic groups has shown strong growth following the pandemic [20], but during recessionary periods, workers of color have been more likely to experience unemployment or leave the labor force [21–24]. Given the impact of gender and race on larger unemployment rates, whether healthcare serves as a stabilizing force for women and members of racially minoritized groups is an unanswered question.

In light of these prior findings, we hypothesize that macrolevel conditions in the economy may have an enduring impact on employment patterns and supply in the healthcare industry that goes beyond the effect of the pandemic. This analysis examines the relationship between the national unemployment rate and transitions of healthcare workers away from providing direct patient care in the healthcare sector (“leavers”) between 2003 and 2022. We expand on prior research by (1) examining healthcare leaver rates over time, (2) quantifying the strength of the relationship between national unemployment and healthcare industry leaver rates, and (3) describing variation in this relationship by type of leaver, occupational and demographic characteristics. The results from this study will further our understanding about healthcare workforce supply trends and how they relate to both economic shocks such as the pandemic and broader economic conditions. Our findings will help organizations anticipate and plan for worker movements under different economic circumstances.

## Methods

### Data and sample

This observational cross-sectional study used nationally representative data from the Annual Social and Economic Supplement (ASEC) of the Current Population Survey (CPS), a complex, monthly panel survey of 60,000 households conducted by the US Bureau of Labor Statistics. ASEC data for 2003–2022 were extracted using IPUMS, a public data service of the University of Minnesota [25]. Our sample included past or current civilian workers aged 18 to 75. ASEC respondents report occupation and industry for the current and prior year for each adult in the household. We used the US Census occupation and industry codes to identify individuals working within the healthcare industry in occupations specific to healthcare. This study was exempted from institutional review because data are fully anonymized and the dataset is publicly available.

### Measures

#### Industry

We used the US Census Industry codes crosswalked to the North American Industry Classification System to define healthcare and selected industries that typically compete with healthcare for workers (i.e., retail, hospitality, educational service, and professional service) [26]. We coded working in the healthcare industry as a binary measure, where a ‘yes’ indicates work in any healthcare delivery settings (Census codes 7970–8290, which exclude drug manufacturers, retail pharmacies, and medical supplies). Within the healthcare sector, we focused our analysis on workers in healthcare-specific occupations involved in healthcare delivery as defined by US Census Occupation codes crosswalked to the Standard Occupational Classification System [26, 27]. We selected these codes of industry and occupation to be consistent with other studies of healthcare leavers [28].

#### Healthcare leavers

Our primary measure of interest was whether an individual left the healthcare industry. We defined individuals as “healthcare leavers” when they reported working in a healthcare-specific occupation within the healthcare industry in the prior year but were either working in a non-healthcare industry, unemployed, or out of the labor force in the current year, and defined “industry leavers” as leavers who were still working (i.e. not retired or unemployed) but not in the health care industry. For example, if a respondent in the 2005 ASEC stated that they worked in a retail setting in the current year of reporting (i.e., 2005) but reported that their prior year work setting was in the hospital as a laboratory technician, then they were defined as an industry leaver [2, 28, 29]. The annual leaver rate was calculated as the number of leavers divided by the number of workers in the prior year.

#### National and industry-specific unemployment rate

We used the Bureau of Labor Statistics seasonally adjusted unemployment rate report to calculate an average annual unemployment rate for the US [30]. We calculated annual unemployment rates for the healthcare industry and selected competing industries from the ASEC data, using the number of individuals in a specific sector reporting a status of “unemployed” divided by all individuals reporting working in that sector in the same current year.

#### Demographics

We used individually reported demographics including gender (available in the data as a binary male/female sex variable) and race and ethnicity, which were together used to create a dummy variably identifying racially minoritized groups that are typically underrepresented in the biomedical workforce (URM), previously defined by the National Institutes of Health to include African American (or Black), American Indian and Alaska Native, Hispanic (or Latina), and Native Hawaiian and other Pacific Islander workers [31]. We used reported US immigration status to look at the percentage of workers who were not US-born citizens.

#### Education level

We collapsed approximately 50 healthcare-specific occupations as defined by the US Census Occupation codes into 9 healthcare occupation groups based on similarities in title and educational requirements, then further collapsed groups by the minimum level of education held by most individuals in the group [32]. A label of ‘high’ indicates a graduate or professional degree (e.g., physicians, dentists), a ‘medium’ label indicates a baccalaureate or associate degree (e.g. RNs, radiology technicians), and ‘low’ indicates less than an associate’s degree (e.g. nursing assistants, home health aides; see table, Additional File 1, which provides specific examples of occupations in each category). Leaver rates for specific occupation groups were calculated by dividing the number of healthcare industry leavers for a given group by the total number of workers in that group the year prior.

### Analysis

Our descriptive analysis examined trends in leaver status for healthcare workers in aggregate, by the state of leaving (i.e., unemployment, retirement, or switching industry), and by selected demographics known to be related to leaving work including gender, URM identity, and education levels. We analyzed the data using Stata/SE 18 including survey weighting functions unless otherwise indicated. ASEC weights are provided with the data to allow for generalizability of the data, and we used the recommended adjusted weights for 2020 to account for different response rates during COVID [33]. We used Pearson’s correlation with weighted estimates to describe relationships between national unemployment rates and industry leaver rates, in aggregate and by education level. We also examined industry leaver rates and unemployment rates in select competitive industries of retail, hospitality, educational services, and professional services. We defined “countercyclical” relationships when trends exhibited a moderate to strong negative correlation at 95% significance [34].

## Results

### Description of sample

The study sample includes 1,790,962 observations of individuals aged 18–75, ranging from 71,214 to 100,262 observations per year, and representing an average weighted estimate of 167.6 million US workers per year (weighted estimates are used from this point forward). Of those workers, a yearly average of 17.7 million individuals reported working in the healthcare industry, or an average of 10.6% of the overall workforce, including 11.7 million in healthcare-specific occupations. The healthcare workforce grew from 9.4% of total employment in 2003 to 11.4% in 2022. Over the study period, gender composition of the healthcare workforce remained stable with 79–81% female workers. Representation by URMs in the healthcare workforce trended upward from 25.3% in 2003 to 30.9% in 2022. The workforce included an annual average 15.2% workers who were not US-born citizens, including a steadily increasing percentage of workers who were naturalized citizens (7.4% in 2003 growing to 11.2% in 2022) and an annual average of non-citizen workers around 5.8% for most of the study period (2003–2019), decreasing to 3-3.5% from 2020 to 2022.

### Description of healthcare leavers

**Fig. 1:**
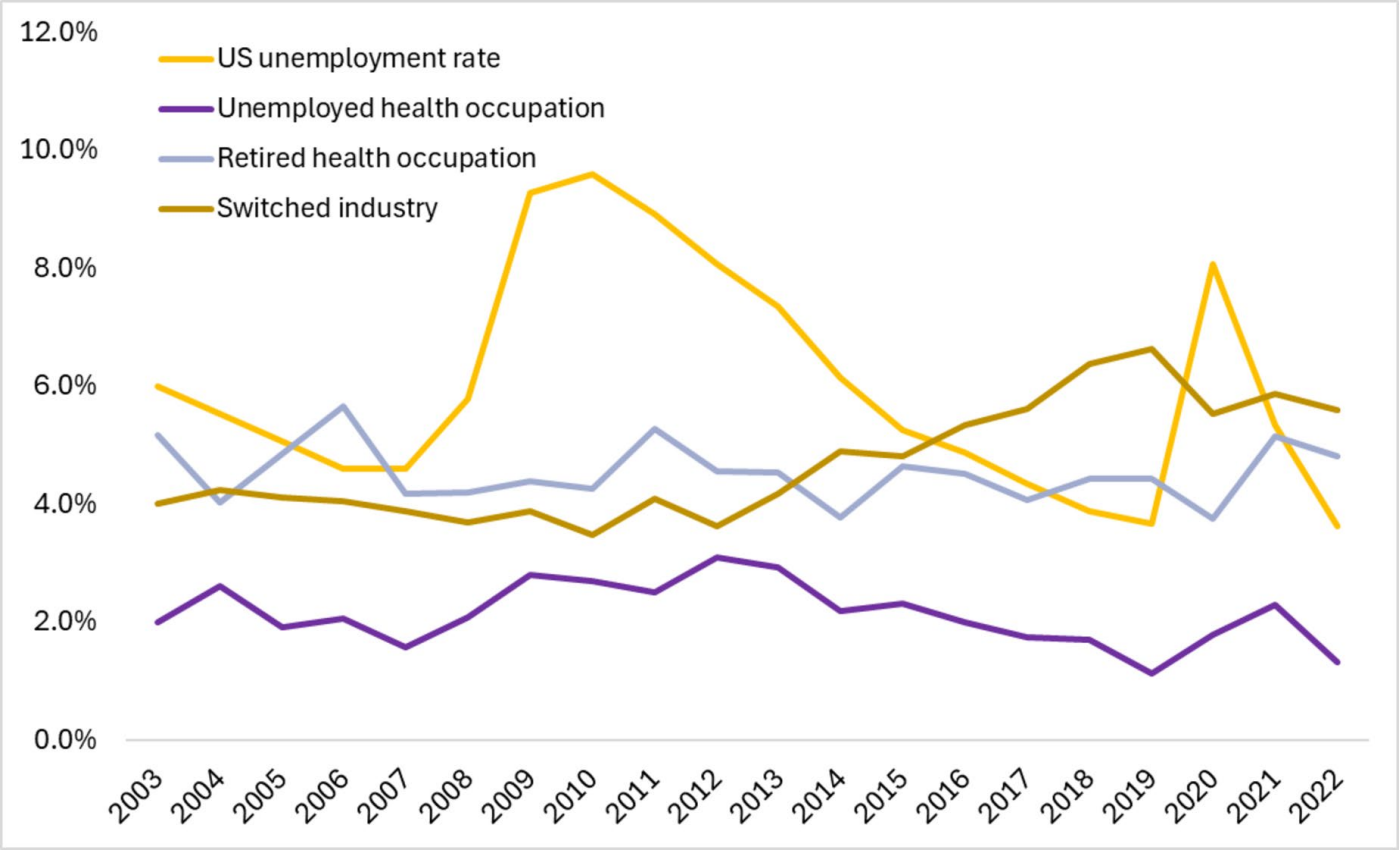
Healthcare Occupation Leavers and the US Unemployment Rate, 2003–2022. *Note*. Rates represent an average annual *N* = 11.7 M healthcare occupation workers in the healthcare industry among *N* = 166.9 M average annual workers in the US. US unemployment rates are drawn from BLS published rates and healthcare unemployment is calculated from the study sample [30]. The ‘left labor force’ category includes healthcare workers who left the labor force for retirement and other unspecified reasons. The ‘switched industry’ category includes workers who reported working in the healthcare industry the past year but working in a different industry in the current year

Our descriptive analysis of trends showed stable rates of leavers across the 20-year study period for healthcare workers moving out of the labor force or to a state of unemployment (Fig. 1). Healthcare workers consistently exited the labor force more frequently than they moved to unemployment (average annual rate 4.5% versus 2.1% respectively). After the onset of COVID-19, healthcare leaver rates increased such that more people were leaving healthcare jobs in 2021 compared to 2020 (13.4% compared to 11.1% inclusive of industry changes, labor force exits and unemployment), but the rates dropped in 2022 to 11.8%.

### Healthcare industry leavers and the national unemployment rate

**Fig. 2:**
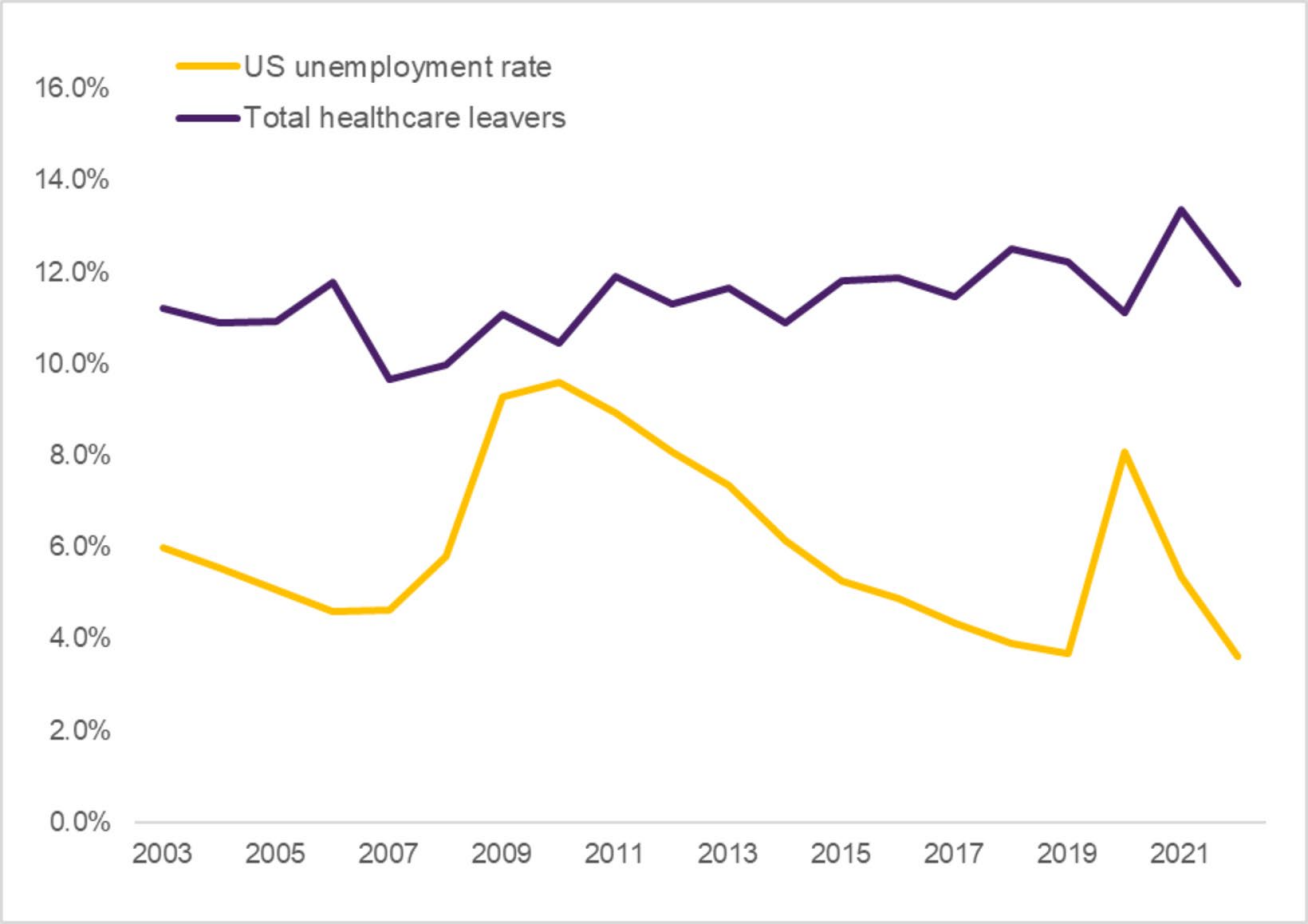
Total Healthcare Leaver Rate and US Unemployment Rate, 2003–2022. *Note*. Rates represent an estimated *N* = 166.9 M workers per year, of which an average 17.7 M are healthcare industry workers including 11.7 M workers in health-specific occupations. Healthcare leavers are defined as individuals in health-specific occupations who report working in the healthcare industry one year prior to the survey but not at the time of the survey, including statuses such as retired, out of the labor force, unemployed, and working in other industries

While the rates of workers leaving the labor force and moving to unemployment remained stable, healthcare industry leavers exhibited more variation throughout the study period. In general, industry leaver rates were countercyclical to national unemployment rates such that fewer healthcare workers left healthcare for a different industry while more workers in the US became unemployed (Fig. 2; r=-0.58, *p*=0.007). Industry leaver rates generally declined in the years leading up to the Great Recession beginning in 2008. During and immediately following the recession (2008/2010), industry leaver rates reached their lowest point (3.5%) while US unemployment reached its highest point (9.6%). As the economy recovered, industry leaver rates steadily reached a peak in 2019 before the start of the pandemic (6.6%), then started to decline in 2021 and 2022.

### Unemployment in healthcare and competing industries

**Fig. 3:**
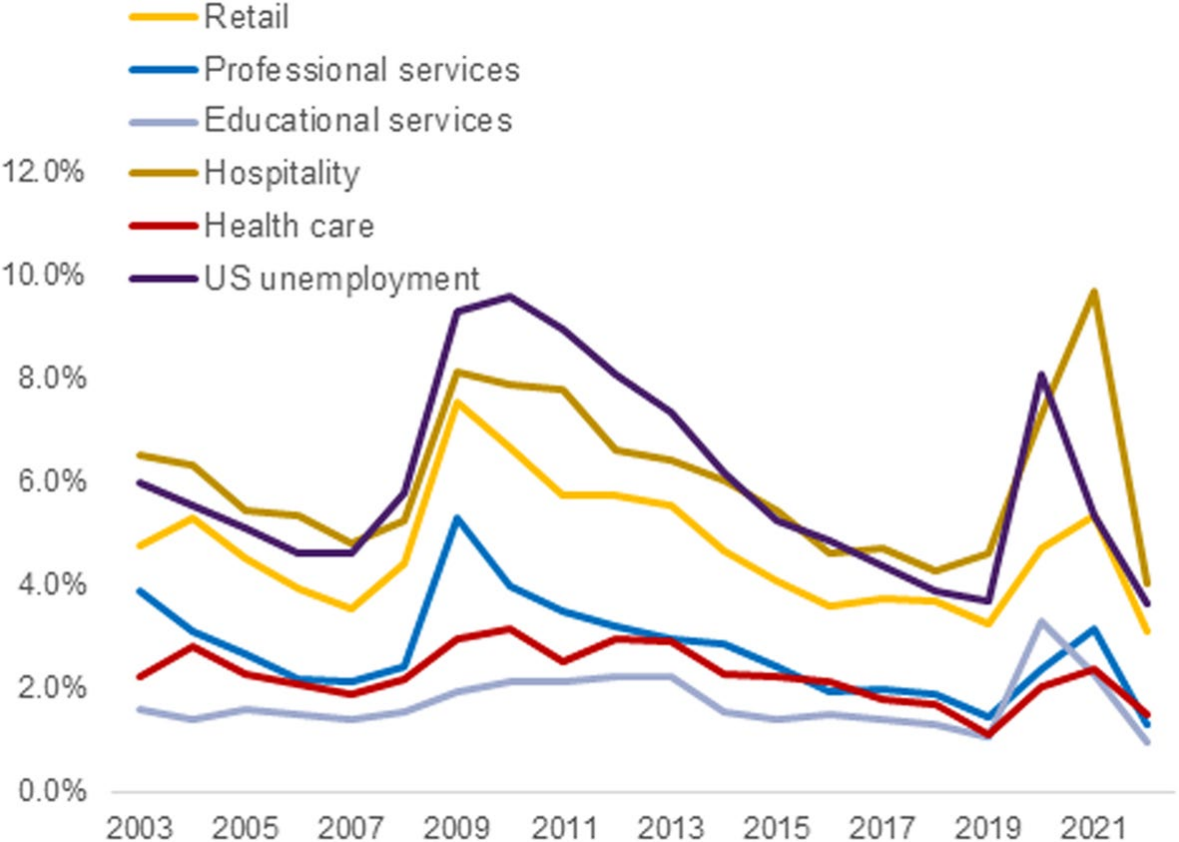
Unemployment Rates in Healthcare and Competing US Industries, 2003–2022. *Note*. Rates show the percentage of workers in a specified industry reporting unemployment and represent an estimated *N* = 3.3B workers in the US during the study period, with average yearly estimates of 18.2 M in retail trade, 11.4 M in professional services, 15.3 M in educational services, 16.0 M in hospitality, and 17.7 M in the healthcare industry (including all workers, not just healthcare occupations)

Healthcare leavers moving into a state of unemployment were not countercyclical to US unemployment (Fig. 3), sharing a similarly shaped pattern with less variability and consistently lower rates overall (range 1.1-3.2% for healthcare unemployment versus 3.6-9.6% for US unemployment). In competing industries, unemployment rates follow the national unemployment pattern, except for educational services, which shared similar patterns to healthcare leavers. Among competitive industries, professional services unemployment and retail unemployment had the strongest negative correlations with industry leaver rates (respectively *r*=-0.61, *p* = 0.005 and *r*=-0.58, *p* = 0.008). There were weak and insignificant negative correlations between industry leavers and hospitality industry unemployment (*r*=-0.29, *p* = 0.215) or educational services unemployment (*r*=-0.21, *p* = 0.375).

### Variation in industry leaver rates across demographic and education groups

**Fig. 4:**
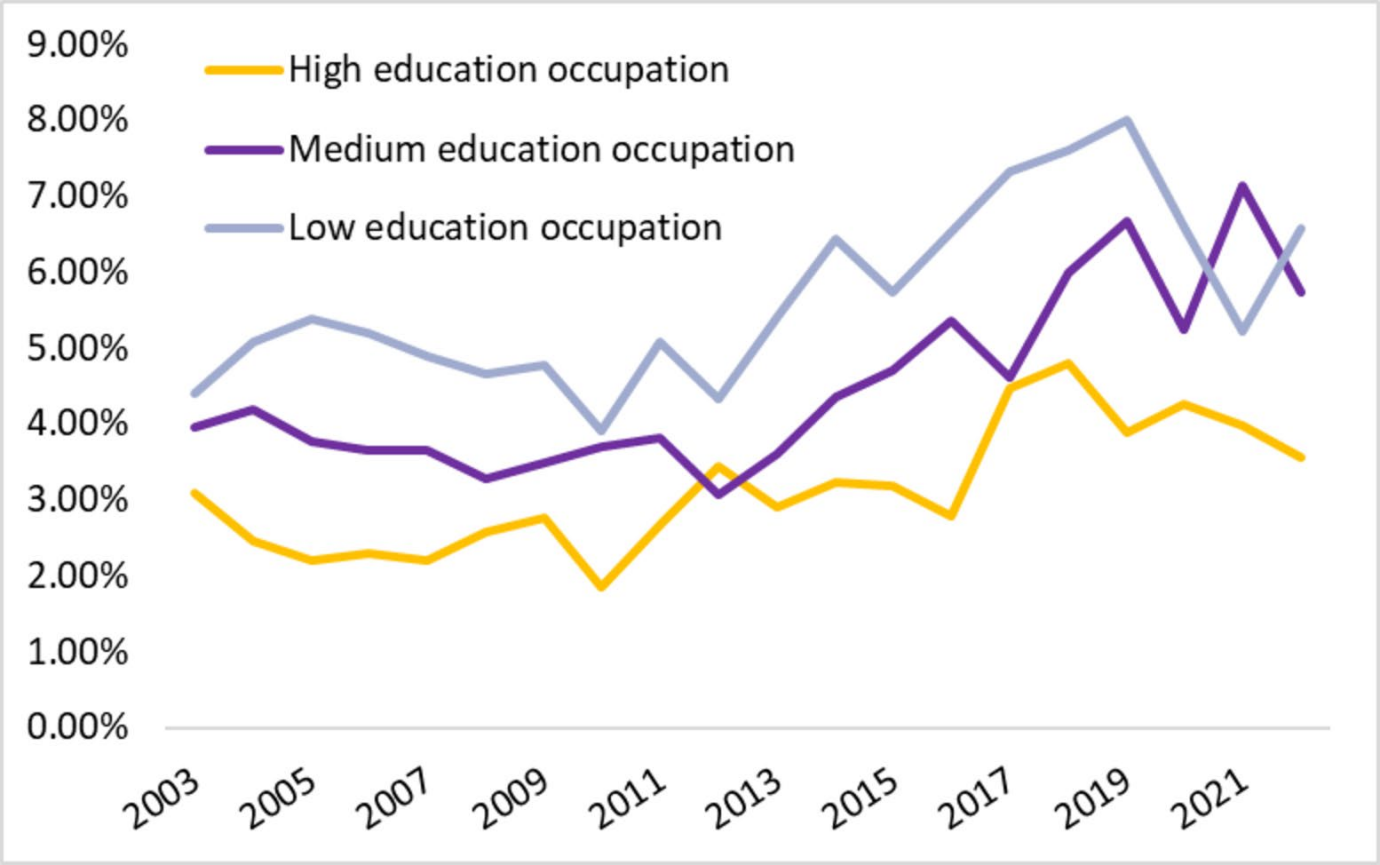
Health Occupation Industry Leavers by Education Level, 2003–2022. *Note*. Industry leaver rates are calculated as the percentage of respondents within each health occupation educational grouping who reported working in the health industry in the year prior but working in a different industry in the year of survey. Health specific occupations represented an estimated annual *N* = 11.7 M healthcare workers over the study period, with average yearly estimates of *N* = 2.1 M, 5.2 M, and 4.5 M for high, medium, and low education occupations respectively

There were no overt differences in industry leaver patterns between men and women in the study period, nor were there substantially different leaver patterns among URMs, although rates tended to be consistently higher with an average of 13.7% compared to 10.5% for white workers. Industry leaver rates for the low education occupational groups (32.2% of the workforce) were highest across the entire period with an annual average of 5.7%, while the medium (50.2% of the workforce) and high education (17.6% of the workforce) groups had lower average leaver rates of 4.5% and 3.1% annually (Fig. 4). The high and medium education industry leaver rates had weak to moderate negative correlations with the US unemployment rate (*r*=-0.32, *p* = 0.169, and *r*=-0.52, *p* = 0.020, respectively), while the low education healthcare industry leaver rate had a stronger significant negative correlation with the unemployment rate (*r*=-0.60, *p* = 0.005).

## Discussion

The COVID-19 pandemic set off a wave of concern about healthcare workforce shortages, with organizations reporting widespread challenges in filling open positions and concerns about a mass exodus of workers [8]. Previous work on post-pandemic workforce shortages has been motivated by a desire to describe or understand the particular impact of the pandemic, which in the current context does not provide the whole picture of macroeconomic forces influencing workforce dynamics. By looking at trends in healthcare leavers over a twenty-year period along with broad economic indicators like the unemployment rate, this study provides critical context for understanding healthcare worker shortages and their relationship to other economic trends.

While the pandemic seemed to be responsible for an initial increase in healthcare industry leaver rates, when put in historical context, we found little evidence that the pandemic triggered wholesale migration out of the healthcare industry. Instead, trends suggest that opportunities outside of healthcare became available to workers, or perhaps workers who made a choice to leave the workforce did so out of necessity. We found that healthcare industry leaver rates were countercyclical to total unemployment rates, highlighting the possibility that current healthcare workforce shortages are as much about broader economic conditions as industry-specific conditions. While future work should more deeply interrogate the nature of this relationship, our results suggest that the much discussed ‘pandemic effect’ on the healthcare workforce may prove to be a less novel ‘unemployment effect’. Ongoing work could also examine leaver patterns of healthcare workers at the state level to ascertain more localized policy shifts and impacts.

Our analysis also provided evidence that this countercyclical relationship was largely unique to healthcare. In several industries that traditionally compete for healthcare workers, unemployment followed overall trends rather than aligning with healthcare trends. This finding suggests that the healthcare industry serves a distinctive role providing safety net jobs in times of economic recession. Previous data suggest that this safety net, at least in specific sectors such as long-term care, functions for workers with all levels of education, drawing some high education workers into areas in which they do not typically work [35]. In our findings, the relationship between unemployment and healthcare industry leavers existed across all healthcare occupations, but it was at its strongest for occupations requiring the lowest level of education. Workers who have fewer educational or credential-based investments may be more likely to leave for competing industries when unemployment rates are low; but in economic recession, they remain in healthcare. These findings show a need to understand the appeal of other industries during a strong economy: is healthcare pushing workers out, or are other industries able to do more to draw workers in?

We did not find evidence of a gender-based difference in either leaver trends or their relationship to unemployment rates. Considering past research on the relationship between gender and employment [18], our findings warrant further research. It may be that the factors that incentivize women to leave the labor market are different from those that shape their choices to change industries. We also know that healthcare is a highly gendered industry, with significantly more women than men. Recent research on the healthcare workforce has focused on the issues workers face, including a lack of available childcare, burnout, and heavy workloads, all of which may push workers—particularly women—out of the workforce [13, 14, 16]. However, our study shows that healthcare jobs may provide women better options and incentives for continuing to work than jobs in other industries. Future work should explore the ways in which gender may not just shape the choice to work but may have a more nuanced influence on career navigation [32].

## Limitations

This study had several limitations. First, the CPS survey noted diminished response rates during the height of the pandemic [36], which we addressed using recommended COVID-specific weighting in our estimates. We also limited our analysis to healthcare sectors and occupations that provide direct care; we did not examine other settings like pharmacies which may compete for workers. Another limitation is that the one year look back provides an aggregated view of worker movements that could mask additional movements in and out of industry or occupation within the year, including those by workers who are not US citizens, making our leaver rates conservative. The analysis looks at very specific sets of conditions such as unemployment or leaving an industry but does not provide details as to causality of job and workforce exits or worker benefits. In looking at macroeconomic trends, we may have missed important difference between states that could be analyzed with larger sample sizes over time. Finally, our descriptive analyses looked at demographics such as gender, URM, and education individually, so we could have missed variances related to intersecting characteristics. This analysis therefore provides a high-level view of job exit among workers in healthcare-specific occupations rather than a granular view of specific groups of individuals or occupations.

## Conclusions

The easing of pandemic-era stresses on healthcare has not ended shortages in the healthcare workforce. The findings of this national, cross-sectional study suggest that when overall unemployment remains low, healthcare worker shortages may be an ongoing issue. The relationship between unemployment and healthcare industry leavers is not unique to the pandemic but is a relationship that has remained consistent for at least two decades. We know that healthcare competes with other industries for workers, particularly workers seeking jobs with lower barriers to entry. In times of recession, healthcare is more successful at keeping workers across occupations and regardless of occupational education requirements. While other research explores interventions aimed at addressing barriers to entry, recruitment, and retention, this analysis suggests that the appropriateness or success of an intervention may depend on broader economic conditions. Healthcare employers should take a long-range, cyclical view of worker shortages by tailoring recruitment and retention strategies to the current economic conditions while planning for the next cycle.

## Supplementary Information

Additional file 1. Detailed education and occupation groups with examples of specific occupations (not exhaustive).

## Data Availability

The datasets generated and/or analyzed during the current study are available in the IPUMS repository, https://cps.ipums.org/cps/.

## Abbreviations

ASEC: Annual Social and Economic Supplement
CPS: Current Population Survey
RN: Registered nurse
URM: Underrepresented in the biomedical workforce
