# Supplementary material for: Beyond the pandemic: the relationship between macroeconomic conditions and healthcare worker shortages in the United States
Source: BMC Health Serv Res. 2025 May 2;25:637. doi: 10.1186/s12913-025-12780-z (PMC12046679; doi:10.1186/s12913-025-12780-z)
Supplement: Supplementary file 1 — Additional file 1. Detailed education and occupation groups with examples of specific occupations (not exhaustive). [file 12913_2025_12780_MOESM1_ESM.docx]

**Additional File 1.**

**Detailed education and occupation groups with examples of specific occupations (not exhaustive).**

| Education group | Occupation group | Examples |
| --- | --- | --- |
| High | Physicians | Physician, surgeon  Doctorate required |
|  | Other healthcare providers (excluding advanced practice) | Chiropractor, dentist, psychologist, optometrist, pharmacist, nutritionist, occupational/physical therapist  Master’s degree or higher required |
|  | Advanced practice providers | Nurse practitioner, physician assistant, certified nurse midwife, certified nurse anesthetist  Master’s degree or higher required |
| Medium | Registered nurses | Registered nurse  Associates or Bachelor’s degree required; few exceptions for diploma RNs |
|  | Therapists | Radiation therapist, respiratory therapist, exercise physiologist, massage therapist, occupational/physical therapy assistant or aide  Associates degree or higher required for ~60% jobs |
|  | Technicians and technologists | Clinical laboratory technicians and technologist, dental hygienist, emergency medical technician, health information technician  Associates degree or higher required for ~50% jobs |
|  | Community workers | Counselors, social workers, health educator, community health worker  Educational requirements vary widely from minimal postsecondary training (home health worker) to baccalaureate or higher (counselors and social workers) |
| Low) | Licensed practical/vocational nurses | Licensed practical or licensed vocational nurse High school degree or GED and postsecondary training required |
|  | Aides and assistants | Nursing, psychiatric, and home health aide, dental and medical assistant, phlebotomist, medical transcriptionist  Jobs require minimal vocational or postsecondary training) |
